# Navigating the Narrative: Integrating Traditional Knowledge and Embodied Practice within Computational Models of Ancient Seafaring

**DOI:** 10.1007/s11457-025-09470-6

**Published:** 2025-09-29

**Authors:** Helen Farr, Benoît Bérard, Joseph Genz, Justin Leidwanger, Emma Slayton

**Affiliations:** 1 Department of Archaeology, University of Southampton, Southampton, UK; 2 Department of History, Université Des Antilles, Martinique, Martinique; 3 Anthropology Department, University of Hawaii at Hilo, Hilo, USA; 4 Department of Classics, Stanford University, Stanford, USA; 5 Carnegie Mellon University Libraries, Carnegie Mellon University, Pittsburgh, USA

**Keywords:** Seafaring, navigation, practice, heritage

## Abstract

Why do people go to sea? The relationship people have with land and sea, maritime space, boats and ships are entwined and complex, shaped and molded by the marine environment, identity and heritage. This paper explores the complexity of people’s relationship with the sea to question how we can understand and model seafaring in the past, and how this can be used to better understand maritime heritage today. To be meaningful, computational analysis of seafaring must be tied into relevant known seafaring and navigation practice. Without this firm basis our statistical and hypothetical models lose the ability to measure past actions. However, there are many ways to ‘go to sea’ and seafaring practices do not start and end on the water itself. This paper reflects upon the process of seafaring, as it relates to our understanding of navigational knowledge, mobility in practice, seafaring as social action and the influences behind people’s desire to set sail. There is much we can learn from applied practices of seafaring, conducted both by practitioners and through efforts of experimental or experiential archaeology; understanding the complexity and nuance of the social aspects of seafaring guides the research questions that shape our models and shapes how we use and understand the outcomes derived from quantitative computational approaches.

## Introduction

Our theoretical approaches shape the questions we ask and the methodologies we use, creating an often-unconscious lens through which results are interpreted, ascribed value, and contextualised within our understanding of the world. As more people turn to computational modeling as a tool to simulate and synthesise complex data and multiple variables to help understand and predict past movement, terrestrial or maritime, it is timely to contemplate how we understand travel and mobility in the past. In regard to seafaring, the focus of this volume, our theoretical approach shapes our computational models, not only ascribing what needs to be included in the broader aspects of constructing maritime landscapes and environments (currents, tides, wind direction and strength, swell, fetch, seasonality etc.), but in our attempt to understand social aspects of seafaring and people’s agency within the process. The actual practice of voyaging is just as complex as the environmental and social landscapes through which people travelled. So too are the methods that we as researchers employ to capture, recreate, or understand seafaring in the past. These include the study of direct archaeological evidence, oral traditions and written records, experimental archaeology and work with modern seafaring practitioners and maritime communities.

Studying mobility in the past is a challenge. Material cultural approaches to understanding past movement and trade have traditionally focused on the start or end points of an object’s journey; elucidating potential directions of mobility but falling short of narrating the process of the journey. This is even more manifest in the case of maritime mobility. In the past, the sea was seen as a barrier to connection, a boundary that separated people on differing coasts, or an empty space on the map: devoid of social activity, if not cultural significance (Ford 2011, p. 763; Flatman 2011). Aligning with ideas of liminality, the sea itself was seen as a transitional space, separating places of action. Conceptualising the sea as a limit to engagement is now considered an outmoded way of thinking (e.g. Blue et al. 2003; Jarriel 2018). Maritime *spaces* have transitioned into *places* of connectivity, dwelling and activity, imbued with meaning and agency. Models help us to understand mobility, they allow us to simulate how people travelled through maritime space, for example, looking at distance, potential routes, viewsheds and lines-of-sight, to help conceptualise the nature of a voyage (Farr 2010a). Computational techniques allow for more complex models, spatial analyses and statistical analyses of ‘digital sea-trials’ that can be analysed alongside any available archaeological data. These computational models have become particularly useful for inclusion of environmental parameters, and for simulating past maritime environments, as well as for testing theories about seafaring technologies in the past. They are a tool for thinking about the process of seafaring within a given space and environment. However, the study of past maritime activity strives to understand the varied and complex ways seascapes were experienced and used. Broader attempts to understand the texture and complex nuance of these seascapes ontologically, and as experienced and created by people, have included hermeneutic, haptic, phenomenological, experimental and ethnographic approaches (Heidegger 1993; Gojda 2001; Ingold 1993, 2021; Steinberg and Peters 2015). Landscape—and seascape—discussions have acknowledged the dynamic nature of place, the temporality of which is created through bodily engagement, with haptic knowledge shaping human knowledge of the world (Ingold 1993; Tilley 2004, p. 3; Farr 2006).

The challenge this sets us is how to incorporate this engaged, entangled and embodied notion of seafaring into computer models of voyaging? Our computational models can be seen as tools for testing and elucidating general ideas and tentative theories. Perhaps then they are not tools that allow for agency and specific nuance?

The much quoted, pithy statement by George Box in 1976 asserts: “all models are wrong” (Box 1976, p. 792) and yet, models do remain useful. For example, palaeogeographical change, in sea-level or coastal geomorphology, can be included in our models of deep-time allowing us to create boundaries for physical land and seascapes (Kealy et al. 2017; Bird et al. 2018; Kuijjer et al. 2022; Borreggine et al. 2022), whilst environmental factors such as climate and oceanographic modeling can help us understand the marine environmental inputs for our models. These allow for an environmentally nuanced understanding of voyaging that can aid our understanding of routing and connectivity. These models highlight why particular routes within maritime space, and particular seasons, may create ‘maritime highways’ for mobility or travel (Irwin et al. 1990; Blankshein 2022; Kuijjer et al. 2022), or by contrast why they might generate barriers that can otherwise be imperceptible at first glance. This is a useful step in understanding past seafaring activity and seascape, but we also need to address how these models can flatten the way we understand people’s relationship with the sea, today and in the past. Box’s (1976, p. 791) legacy was not just the critique of all models, but rather the advocacy of advancement of knowledge through multiple iterations between theory and practice within a feedback loop. Thus, with awareness of modeling limitations, we can return to theory (and the practice of seafaring itself) to inform new and improved iterations of the models we produce.

In order to construct useful models, we need to understand the ways in which the sea can be used and understood, and somehow incorporate the intellectual, spiritual, emotional and physical realities of mariners’ lives within them—no small task. To understand a person’s experience of going to sea, their choices, their desire, their perception of risk, is hard enough today, let alone in deep time. These social aspects of seafaring are specific and intrinsically tied to time and place. Yet as modelers, these parameters affect our chosen inputs and shape our understanding of what is feasible, as well as providing us with broader contextual understanding to allow for better evaluation of our model outputs.

A deeper understanding of the practice of seafaring aids our own comprehension of its scope beyond a simple movement between ‘A’ and ‘B’ within a marine environment. Whilst the processes involved within this sphere of activity may be generalised, voyages cannot. Seafaring is a local practice that can occur on a global scale. The reasons why people go to sea and the experience of it differ from region to region, community to community and individual to individual.

## From Theory to Praxis

Applying various archaeological theories and methodologies to the understanding of past seafaring practice—computer generated or otherwise—is vital if we are to develop questions that delve into the realities of seafaring. Computational modeling is best used to inspire further inquiry rather than provide absolutes, and the exploration of real-world conditions is essential in making these lines of exploration meaningful. Tension between maritime realities and computational capabilities derive in part from the limitations of model inputs—such as understanding forecasted climate data, limited archaeological and ethnographic information, and technological recreations. Applying theoretical approaches based on what we *do* know about seafaring practice in today’s world can help researchers to better evaluate and attempt to validate computational outputs.

Theoretical perspectives are vital building blocks for modeling as they help to center the human condition and experience within a digital framework that often relies on fixed absolutes of environmental or geopositional data. In some sense, we hope that thinking around seafaring practice adds value not only to the archaeologist exploring questions of seafaring, but also to prepare those from computational backgrounds to fully recognise the practical, social and emotional components of seafaring alongside the physical and technological limitations. By identifying the issues of concern to seafarers past and present—such as how they interact with their environment or prepare for the journey—we begin to embody seafaring practice and recognise the nuance needed within these models.

During the CAST workshop in 2022, it became clear that a strong foundation in anthropological theory and ethnographic research directly impacted many issues relating to the practical application of modeling or archaeological seafaring research. Further, it became apparent that every contributor felt there was a need to include discussion of, reflection on, and inclusion of the experiences and needs of those who would have made journeys across the sea. Though the theoretical frameworks we employ in our work vary, there is a shared key focus on (A) people’s engagement with their environments, (B) understanding maritime technologies, and (C) exploring traditional knowledge and what it can uncover about past practice.

As we approach these questions, it is crucial to identify the various approaches researchers take to understanding past practices. Even amongst the authors of this paper, or more broadly, this volume, there are several approaches to exploring the act of seafaring and all that it entails. That there are so many different modes of knowing, or approaches to data collection, demonstrates the richness and complexity of maritime communities’ relationship to the sea and highlights the need to avoid generalised normative narratives. Various approaches include marine and earth science, anthropological and ethnographic theory, traditional knowledge practices, historical sources, the archaeological record and experimental archeology. Evaluating past navigation practices, or the process of moving across the sea in a regular pattern, requires the interweaving of all these fields. Information on the location of the beginning and end of journeys may come from the archaeological record, whilst the actions of people to share information on where the destination *is* may come from ethnography or shared practitioner knowledge. Tracking the process of human decision making in the past is difficult, but crucial to the development of practical computational models that require the virtual agent to make ‘decisions’ based on pre-selected inputs that evaluate both environmental data as well as response to these changing stimuli weighted against preference to travel in specific directions. Understanding how these approaches can work together can enable researchers to explore ways to use multiple sources of information to reconstruct not only the sea-routes travelled in the past but the seafaring practices that ensured their success.

This broad and interdisciplinary approach to seafaring research is necessitated by the wide range of geographic contexts to be studied and the range of environments that are included in the practice of navigating between two points. Though many computational models often focus solely on the ocean surface as the plane of movement, practitioners have highlighted the importance of viewing their travel space as happening in a wider ‘maritory’: a broader, contextual space in which seafaring happens or is discussed (Needham and Clark 2009, p.12; Fleury 2013; Barrena et al. 2022). Reflecting on maritories and the maritimity of a community can highlight the need to embed and interweave narratives of seafaring practice into wider discussion on place, cosmology and identity.

To take up a richly narrative, particularising, maritory approach to aid thinking through seafaring as social action, this paper metaphorically undertakes a voyage. This echoes the narrative tradition within Pacific Island Studies, where the voyage is a common metaphor within storytelling and teaching (Teaiwa 2005). The piece is an amalgamation of examples from researchers focused on different chronological periods and geographic areas of study, from the Mediterranean to the Pacific and the Caribbean. This provides different examples of seafaring practice; it does not seek to generalise all seafaring practice, but to highlight the rich ways voyages can be socially embodied and embedded within community. Our goal is that by splitting our evaluation of human action into the separate stages of a voyage, it may be easier to understand both the *when* and the *why* of human action in maritime spaces, and to apply relevant theoretical frameworks that can address these choices within the context of real-world practice, not just within computational constraints. Here, we discuss the process of a voyage from: *planning a journey* (addressing the complexities of voyaging goals and how we can identify past planning), *building a boat* (discussing seafaring tool kits and their impact on sailing and community networks), *preparing a journey* (analysing community actions that allow for both short or long voyages), *going to sea* (recognising the social challenges, emotions and opportunities of being at sea mid-voyage), and *recounting the voyage* (evaluating navigation techniques and cultural practices for sharing the stories of being at sea). In many ways these sections are also reflexive, allowing us to consider our role as computational researchers within this maritory space. These sections will relate to other papers in this volume that more directly address practical applications of these activities in current research but also serve as a primer for what theoretical backgrounds should be explored prior to turning on a computer to build hypothetical-computer-generated routes.

Today, the co-authors of this paper each metaphorically ‘head to sea' in their research for various reasons; motivated to learn about past seafaring practices using methods grounded in the co-production of knowledge and to work with cultural practitioners to fulfil community-based goals. In this way this work highlights seafaring heritage, both tangible and intangible, past, present and future.

### Why do People go to Sea?

As archaeologists we often talk of ‘push’ and ‘pull’ factors as the motivators for people to step off dry land, to *become* seafarers, yet these short-hand terms mask complex decision making. Not all seafarers choose to go to sea, and some have little choice; when we consider the push factors that result in forced migration, for instance, ‘going to sea’ may reflect a *lack* of other choices. However, even within more routine seafaring practice, the decision to ‘go to sea’ is never simple. There are many communal needs or social goals that are weighed against practical and technological factors before a voyage can be made.

Looking back to the origins of seafaring in deep-time, a fundamental question remains why we, in our remote past, went to sea? There are many diverse reasons for seafaring. In some parts of the world, turning to the ocean involved exploring, discovering and settling new islands and lands, with various “push” factors such as population pressure, over-exploitation of critical resources and climate disasters, and “pull” factors such as unique island resources, or a ranked social structure where status could be gained from seafaring, or where older siblings inherited titles to land, which may have incentivised younger siblings to discover new lands to claim as their own as well as voyaging as an exciting adventure (see Finney 1986 for a characterisation of heading to sea in Oceania’s deep-time). Voyaging over longer distances may also have been dependent on, or even an outgrowth of, earlier tentative steps into the water to harvest marine resources. Even the Mediterranean Sea’s comparatively small size and short distances amongst (mostly) visually connected lands generated diverse patterns of movement by our earliest ancestors. Maritime activity aimed at securing food and raw material resources, and also facilitating exogamy, occurred against a backdrop of changing climate and radically shifting coastlines over several hundred thousand years (Broodbank 2013). A mix of motivations and technologies would have likely impacted decisions about driving the earliest seafarers to explore and settle distant, uninhabited worlds, as well as their descendants who continued to ply the seas along known routes.

Maritime exploration within Island Southeast Asia and Oceania provide key case studies into the motivations behind early seafaring, where the first exploratory steps by early humans to shift from terrestrial migrations to ocean crossings began 50,000–65,000 years ago during the Pleistocene (Kirch 2017; Norman et al. 2018; Ditchfield et al. 2022). With climate-induced lowered sea levels and exposed continental shelves, these early seafarers left their known world on the Sunda shelf (what is now Island Southeast Asia) to reach the supercontinent of Sahul (the connected landmass of Australia and New Guinea) when sea-levels were lower during Marine Isotope Stage 4 (Kuijjer et al. 2022). Archaeological excavations of rock shelters on the Wallacean islands, northern Australia, New Britain and New Ireland suggest that these Pleistocene mariners developed some form of watercraft such as bamboo rafts, bark boats or dugout canoes. Whilst there is no direct archaeological evidence for these voyages, computational modeling, allowing for a complex meshing of palaeogeographical and environmental factors, and a high number of test-runs, has begun to answer questions as to these earliest maritime technological developments, routing, duration and propulsion (Bird 2018; Norman et al. 2018; Kuijjer et al. 2022).

These early mariners reached the eastern edge of the Solomon Islands by 20,000 years ago, but here the first phase of human oceanic expansion is currently thought to have ended. It was not until about 3500 years ago—with near-current sea levels of the Holocene—that Austronesian seafarers developed forms of watercraft and navigation techniques that enabled deep ocean voyaging and long-distance exploration that eventually led to the discovery and settlement of the islands of Oceania (Genz et al. 2023).

Whilst the Pacific Ocean may be described as the birthplace of true ocean voyaging, it is also the birthplace of many computational seafaring models. Simulations largely converge with historic accounts, oral traditions and insights from experimental canoe voyaging on the strategic use of weather patterns that supports the idea of systematic exploration strategies—simulations of thousands of voyages setting out from various islands result in some one-way accidental drift voyages or navigational errors that contributed to the inadvertent discovery of islands, but such a scenario cannot account for settlement of all of Oceania (Irwin 1992, 2007). The techniques employed in these exploration strategies, and replicated in computational models, largely depended on the technological toolkits of those headed out to sea as well as oscillations in climate and ENSO patterns effecting rainfall (Sear et al. 2020; Dickson et al. 2019). Smaller, paddled, human-powered vessels would have needed to check the direction of flowing currents that have often dictated the direction of travel (Callaghan 2001; Slayton 2018). Wind strength and direction would have affected all types of watercraft, not just those with sails. Sailing vessels are proposed to have searched safely in an upwind direction against the direction of prevailing easterly trade winds by tacking against the winds or by exploiting seasonal westerly wind shifts, and then (whether they found uninhabited islands or not), returning home with the resumption of the trade winds to embark on future expeditions (Finney 1985, 1986; Irwin 1992, 2007).

The early voyages of the Pleistocene and later Austronesian expansion in the region of Oceania provide insights into the nature of our earliest seafaring practices, centered on the question of why people go to sea as well as understanding subsequent voyages. For the surviving descendants of those intrepid explorers, and among other Indigenous maritime communities throughout the world, the question shifts to more of an ontological framing. The Tongan anthropologist Epeli Hauʻofa (1998, p. 58) wrote in a seminal essay, “…the sea is our pathway to each other and to everyone else, the sea is our endless saga, the sea is our most powerful metaphor, the ocean is in us”. Drawing from Hauʻofa’s vision, historian Paul D’Arcy (2006) explored the intimate connections that “people of the sea” have with the ocean, where canoe travel is part of everyday life, and belief systems and language are deeply embedded within concepts of the sea. In the Marshall Islands, for example, an oral tradition of cosmogonic origins describes how at the dawn of time, the fabric of the universe was a watery expanse,where in the opening of a giant clam shell by deities resulted in the creation of our world, the genesis of islands, and the first people (Genz 2018, p. 25). The Marshallese, who identify as being born from the sea, have encoded this idea linguistically, with the term for navigator (*ri-meto*) meaning “people of the sea” (Genz 2018, p. 1). Narratives and oral traditions that tell of origins from, or from across, the sea, are common in many different parts of the world.

Beyond consideration of the sea being a liminal space, and an intrinsic component of identity for many maritime focused peoples, the sea is also a resource for both sustenance and community engagement. Indeed, it can be helpful to consider the canoe as a mobile site, or a space of both community engagement and practice. While regionally specific, in 2022 a group comprised of scholars and cultural practitioners convened a workshop at the Center for Pacific Islands Studies at the University of Hawaiʻi-Mānoa to share storied experiences and scholarly research to co-write about the histories and contemporary practices of current seafaring in Oceania for the Teaching Oceania Series (Genz et al. 2023). One of the guiding frameworks that emerged from the workshop was that for Indigenous maritime communities the canoe is simultaneously a vehicle for forging life-sustaining local and regional exchange networks, and evidence of a thriving community and culture in terms of healthy social relationships; a vibrant forest ecosystem with a wealth of resources, and well-established generational thinking with innovative mindsets. Thus, after the initial period of exploration, discovery and settlement, Pacific Islanders remained highly mobile, regularly voyaging among and across islands and archipelagoes.

**Fig. 1:**
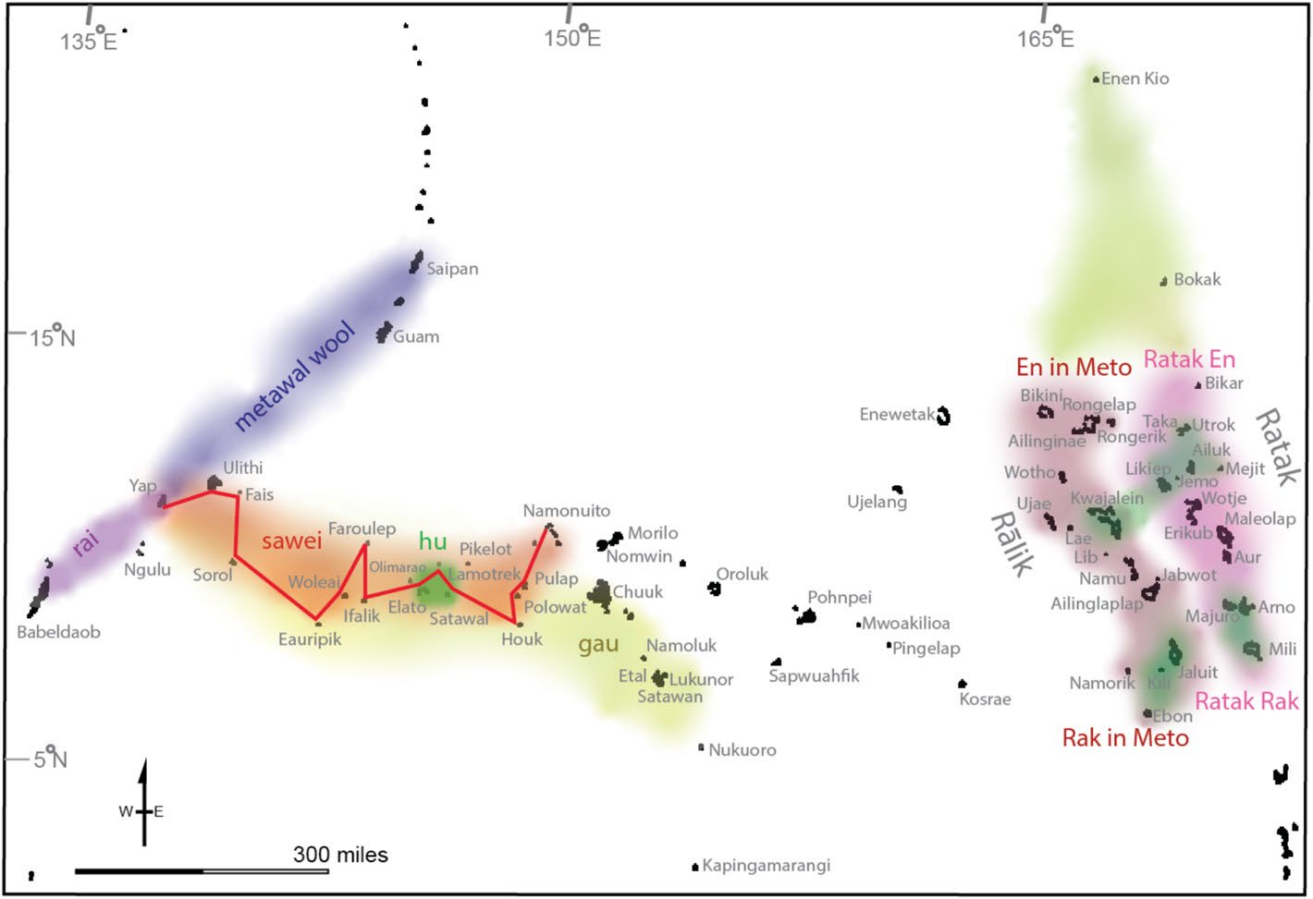
Interaction spheres of Yap and the Outer Islands (left) and the Marshall Islands (right), described in Tamagyongfal et al. (2023, p. 55, Fig. 5)

Voyaging in “a sea of islands” (Hauʻofa 1994) afforded food security and maintained exchange networks that provided access to resources, technologies, valuables and knowledge. Drawing from archaeological artefacts, historical linguistic reconstruction, and oral traditions, scholars and practitioners describe several post-settlement voyaging interaction spheres in Oceania (Feinberg 1991; Genz et al. 2023). The *Vasa Loloa* centered on a three-way exchange of canoe wood and red bird feathers from Fiji, tapa cloth from Tonga, and fine mats and tattooing from Sāmoa (Davenport 1962, 1964). The *kula* linked the communities in the Massim archipelago in southeastern Papua New Guinea in an exchange of symbolic items (necklaces and armbands) that enhanced social status and prestige. An interaction sphere linked the high volcanic island of Mangareva in the Gambier Islands with nearby islands in marginal environments that supplied high prestige resources (red feathers, turtles, particular stones) (Davenport 1962; Molle and Hermann 2018). The *sawei* linked Yap to islands as far east as Chuuk in a system involved gift exchange, religious and political tribute, and disaster relief. And in the Marshall Islands, linguistic homogeneity extends farther than anywhere else in Oceania—encompassing 750,000 square miles—as a result of extensive, regular interaction including networks that linked atolls in the dry north with long-term inter-annual variability in rainfall and drought with atolls in the wet south (Tamagyongfal et al. 2023) (Fig. 1). These and other voyages of interaction demonstrate continued communication between the home islands and those islands newly settled; some of these networks ceased prior to European colonisation, some operated until the historic era, and certain aspects of the networks have endured—embedded as values placed on social relationships—even though the canoe voyages have largely stopped (see Genz et al. 2023 for a detailed examination of the post-settlement interaction spheres of Oceania).

Whether considering the first tentative journeys and the origins of seafaring, or the more recent complex networks, consideration of *why* people become seafarers is closely linked to need, whether social, environmental or economic. Within our computational seafaring models, our assumptions may be reduced to the desire to test out early colonisation movement across the sea (Kuijjer et al. 2022), or to explore connection between known sites. In both cases, it is imperative to ask these questions to better understand technology, direction and routing and the next stages of planning.

### Planning a Journey

As we have just seen, there are many reasons why communities have turned toward the sea and sustained seafaring traditions, over generations and sometimes for thousands or even tens of thousands of years. The processes involved in the decision-making and implementation of these initiatives are only anecdotally the work of a single person. Building a boat, or organising an expedition, is usually part of a complex collective process. In varying proportions, these plans derive from an interweaving of social, political, economic and symbolic (religious) factors (see Shaikh 2022, p. 1). Access to these mechanisms is particularly challenging, if not impossible, in the absence of historical sources or direct ethnographic data. Conversely, when we have access to such information, we can recognise its richness and complexity.

Diverse motivations frame the particular planning behind any journey, and each driver dictates the individuals involved, the cargo or other load carried, the type of vessel (and whether new or old), the route, and the balancing of environmental variables along with risk and reward. For example, seafaring in search of marine or other resources will privilege certain vessels, and even the specific properties of the resources themselves will in turn necessitate certain cargo capacities, speeds and timelines, journey sequences, labor skills and allocations, traditions, and other considerations that are themselves entangled: to cite just one example, seasons of journeys are tied to the feasibility of making a voyage within the weather conditions, as well as the availability of the resources sought but also the potential seasonal availability of material and labor resources. Regular exchange and communication demand different parameters for journey planning, and the challenges of large-scale migration surely did also. This is not to say that these journeys are unrelated in their planning, and certainly individuals, knowledge, techniques and even vessels are shared, but the many and varied rationales behind a journey must be acknowledged and then the demands entailed must be articulated in our approach if our modeling is to go beyond the superficial and be embedded deeply in practice.

On the other hand, when the primary motivations behind different past journeys are transparent, we still only get a faint glimpse of the actual processes of planning they entailed. Even the particularly detailed texts from the ancient Mediterranean offer only scattered snapshots of this process, from courtroom speeches capturing when a journey did not go as planned, to contracts for the arrangement of boatbuilding and trading ventures. One document preserved on a scroll, the so-called Pech Maho lead letter, tells of the partnering of different individuals and relatives in the acquisition of small boats and their cargos, of the roles of social status and ritual dynamics in such negotiations, and of the pledges, investments and sureties that entangled relations across cultural, linguistic and geographical boundaries (Lejeune and Pouilloux 1988; Chadwick 1990). Yet the specifications of the ship itself remain unclear, as do the sources for the provisional cargo and the anticipated destination, not to mention the facets of loading, supplying and crewing. Yet this range of considerations was integral for what, in all likelihood, was still a comparatively simple and routine journey for its period and place. Despite such rare and tantalising windows into the planning of a voyage, much more of the communal planning remains hidden from view, forcing us to rely on conjecture, analogy or experimentation, particularly when faced with such historical distance. Interestingly, experimental archaeological voyaging projects can aid in our comprehension of some of the details of the planning processes of past voyages, whilst the details may be anachronistic, consideration of seasonality, tide times, weather forecasts, crew numbers and roles, and provisioning, give us relevant proxies.

These considerations in planning make collaboration amongst different communities (in the case of experimental voyages, communities of practitioners and researchers) a key framework for successful design and resourcing of journeys. Efforts to design and use a vessel depend on this collaboration.

### Building a Boat

Once the decision has been made to go to sea, the next step is to build a boat. The latter is at once a machine that floats and moves, an instrument linked to a specific function (fishing, cargo, interaction etc.) and a temporary living space for its crew and possible passengers (Muckelroy 1978, p. 221). Thus, a boat is an expression of a technical, cultural, social, economic and even political system. These different functions, the needs to which the future boat or ship must respond, determine its characteristics (Pomey 2013; McGrail 2009; Adams 2013, pp. 15–32). Its construction process is then the result of a dialectical relationship between a technical system and the environment in which the construction phase (particularly in terms of access to raw materials), as well as future navigation (the physicality of seascapes), will take place.

In this way, our computational approaches to recreating voyages echo real life. A central step in the process of computing ancient seafaring is the selection and modeling of a boat, which must be fed by upstream work aimed at reconstructing ancient boats and documenting their seagoing capabilities. Our knowledge of these vessels and their use is based on both archaeological and historical documentation and ethnohistorical and anthropological data combined with marine engineering and knowledge of technology and environment (Dickson et al. 2019). Even when archaeological finds provide researchers with detailed accounts of boat or ship technology, reconstructing the precise nature and construction processes of watercraft generally requires a multidisciplinary perspective.

In order to reconstruct the characteristics of these ancient vessels, numerical data including general size and architecture, cargo-carrying and navigational capacities needs to be collected. This stage involves the creation of a reconstructed vessel. This can be full-scale, in the form of a physical replica, a scaled model for tank testing, or simply a digital twin. It is important to keep in mind that the latter will always remain a floating hypothesis up to a certain point, given the limitations of direct ancient data mentioned above. Tank testing analyses (hydrostatics, hydrodynamic studies) and experimental approaches in the real world can then be developed based on this hypothesis. Since the first Kon Tiki expedition, the interest and limitations of reconstructing boats and practicing experimental maritime archaeology have been the subject of much discussion and the source of extensive literature (e.g. Ravn et al. 2013). Whatever the real limitations of this type of approach (Cherry and Leppard 2015), it gives us more direct access to the technical details of ancient seafaring, allowing us to generate the data needed to produce computational seafaring models. This type of program works best if it is grounded in an exchange between researchers and practitioners from maritime communities.

**Fig. 2:**
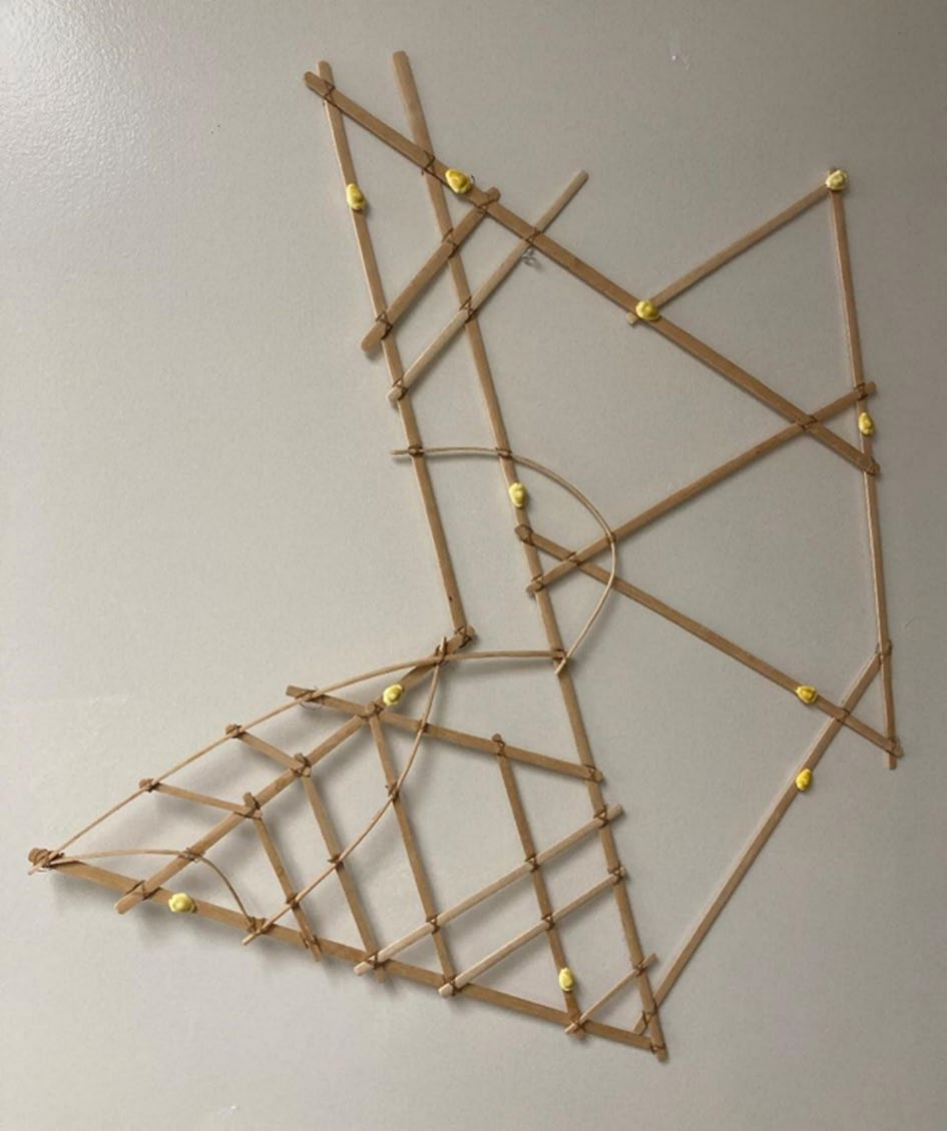
A *meddo*, constructed by Isocker Anwell in 2016, based on a diagram by Schück (1902, Fig. 31) of a Marshallese stick chart collected by Gulick and acquired by the Bernice Pauahi Bishop Museum in Honolulu, depicting the southern region of the western Rālik chain of the Marshall Islands; the cowry shells indicate atolls and the latticework represents wave patterns that afford navigational information (Photo by Genz)

As an example of this articulation between archaeology and history, anthropology of technology, reconstitution of ancient boats, experimental maritime archaeology, and computational modeling of seafaring, we will briefly focus on the example of experimental seafaring approaches in the Caribbean and the question of the seafaring capacity of Indigenous Antilleans (see Fig. 2). This program, while not to be considered exemplary (perhaps like any of its kind), is a good example of the type of process we wish to highlight here.

Questions of seafaring and navigation have obviously always been important in the study of the human settlement process of the Antillean archipelago and in understanding the dynamics of its precolonial occupation. They have become even more so in the last two decades, as connectivity and archipelago have become the central paradigms mobilised by the archaeological community to describe them. In response to this need, a significant scientific effort has been developed over the last decade or so, particularly within the framework of a multidisciplinary collective research program led by one of the authors, which began with a synthesis of archaeological data and historical sources on Amerindian navigation in the archipelago (Fitzpatrick 2013; Bérard et al. 2016a). This aided the description of a variety of vessels (floaters, rafts, dugout canoes, expanded and extended dugout canoes—for definitions, see Arnold 2018) that constituted the fleet of these populations and highlighted the *chaîne opératoire* for the construction of the kanawa of the West Indian Kalinagos in the sixteenth and seventeenth centuries. The results of this work provided the specifications for a project to rebuild one of these canoes, a project that was carried out in collaboration with marine carpenters from the Kali’ña community in French Guiana. The technical process of construction by the carpenters was the subject of an ethno-archaeological study, and the project was completed, at the request of the community, with an oral survey aimed at reconstructing the place of these boats and seafaring practices in the historical and contemporary culture of the Kali’ña (Bérard and Biar 2021). Smaller-scale work was also carried out with the *gommiers* (dugout canoe) builders of the Kalinago territory of Dominica. In this way, several kanawa were reconstructed by interweaving archaeological and historical data with the technical traditions of the last sea dugout canoe builders in the West Indies and French Guiana.

The second step was to evaluate the capabilities of these boats, one of which was also first modeled in 3D, and this formed the basis for the development of a hydrostatic study (Billard and Bérard 2009). This allowed different loading hypotheses to be tested and their impact on both the speed and stability of the boat—a crucial issue for sea-going canoes without outriggers—to be assessed. Finally, the "Akayouman" kanawa was used on various coastal navigation and inter-island expeditions within the Antilles archipelago as part of a participatory experimental maritime archaeology program based on the mobilisation of various actors from the Amerindian and Creole communities of the Antilles (Bérard et al. 2011, 2016b). The data collected in this way has allowed us to broaden the discussion on the settlement of the archipelago and the navigational capacities of the pre-colonial Indigenous populations. Finally, all the knowledge generated by this program has contributed to the development of a numerical simulation program (Slayton 2018), which in turn has led to new advances in the computational modeling of seafaring more generally (Hofman et al. 2019, 2021).

Knowledge of the physical characteristics of boats and how they affect their sailing performance is clearly a key factor. However, it is only one part of the complex system of interactions on which seafaring is based, and only one of the elements to be considered when preparing for a voyage.

### Preparing for the Journey

Preparations for going to sea include consideration of when and where to go and how to get there, in addition to what particular knowledge, technology or things are required along the way, or on arrival. These would have differed through time, and reflected the reasons for going to sea and the available technology or distance.

In the Pacific, detailed ethnographies on seafaring practices and traditional systems of wayfinding in Oceania illuminate navigators’ preparations and skill, including mental processing of environmental phenomena and embodied knowledge of the ocean. Studies with navigators from Polowat (Gladwin 1970), Satawal (Thomas 1987), Indonesia (Ammarell 1999), Anuta (Feinberg 1988), Taumako (George 2012), and the Marshall Islands (Genz 2018), coupled with in-depth studies on traditional navigation in Oceania (Howe 2007; Finney 1998; Lewis 1994), illuminate culturally-specific navigational toolkits that apprentice navigators learn over the course of years in their youth. This preparation for voyaging includes both land-based instruction and experience at sea (Pyrek and Feinberg 2016). Recognising the complementarity of mental models and embodied knowledge (Genz 2014) along with the limitations of language to access these ways of knowing (Feinberg and Genz 2012), researchers of navigation identify three analytical divisions of navigation—setting and maintaining a course, estimating position once out of sight of land with consideration of leeway and currents, and homing in on the target island.

Two case studies—Polowat and the Marshall Islands—illustrate the cultural elaboration of a general system of Pacific navigation through the lens of anthropology. Thomas Gladwin’s (1970) pioneering ethnography of Polowatese navigation detailed how navigators invoked and integrated multiple schemas, including learning conceptual compasses for spatial orientation and course-setting based on wind directions and the rising and setting positions of stars and other celestial objects (the “star compass”), a moving reference island concept (*etak*) to estimate position and keep on course through dead-reckoning procedures, and various concepts to expand the range at which islands can be detected in order to make landfall: such as cloud formations, flight patterns of birds, and disrupted swell and current patterns. Lewis (1994, p. 196) demonstrated that Pacific navigators only need to get close enough to a target island so that it can be detected remotely through these various land-finding techniques, which typically means 30 miles for a low-lying coral atoll and 50 miles for a high volcanic island.

Navigators in the Marshall Islands developed a system of wayfinding focused on remotely detecting how islands disrupt the flow of swell and currents (Genz 2018). Marshallese navigators pilot a canoe through embodied ways of knowing, feeling how waves affect the motion of the canoe, which in turn affords navigational information in terms of distance and direction toward an island (Genz 2014). Marshallese navigators developed “stick charts” (*mattang* or *wapepe)* that abstractly model the conceptual framework underlying wave navigation, including Indigenous concepts that partly resonate with Western oceanographic understandings of wave reflection, refraction and diffraction. Navigators also developed planar representations to map the real positions of atolls and ocean conditions, including regional cartographies (*medo*) (Fig. 2) and maps of an entire island chain or the entire archipelago (*rebbelib*). The modeling and mapping of these Indigenous teaching devices are material representations of the navigator’s embodied knowledge of the sea (Genz 2016).

With this knowledge, navigators must consider the climatic conditions—local weather and anticipated seasonal shifts in weather systems—and island geographies in relation to canoe technologies and designs (see Gladwin 1970 and more recently Dickson et al. 2019). Irwin’s (1992, 2007) thesis of systematic voyaging (see above) is also based on this learning and knowledge and describes the relative safety of sailing upwind, in order that boats can easily return home without being required to tack back and forth against the trade winds. However, seafarers from Polowat and the Marshall Islands developed lateen-rigged shunting outrigger canoes that are designed for stability travelling on a beam reach across the wind, maintaining the outrigger on the windward side of the canoe. Thus, most routes in the Marshall Islands, for example, travel north and south within each island chain (the eastern Ratak chain and the western Rālik chain) with the predominant easterly trade winds remaining on a boat’s beam, rather than travelling east–west between the island chains. Specialists in weather forecasting prepare for the voyage by staying attuned to the environment through daily observations and track the seasonality of preferred winds and storm systems. The relative calm period of the summer months in the Marshall Islands, for example, is interlaced with storms predicted by the presence of certain stars marking the annual passage of time, but summer travel is still preferred to voyaging in the extreme windy months of winter in which the wind-driven sea state masks the more subtle wave patterns used in navigation (Genz 2018).

Complementing this anthropological understanding of the sorts of knowledge and preparation for navigation are the Indigenous and practitioner understandings of wayfinding. As the keynote speakers to the CAST workshop describe (see Raigetal and Kelen, this issue), there are social, political and spiritual elements that drive their understanding of the sea and inform their decisions about how to employ navigational knowledge, strategise about specific routes and determine the optimal timing of the voyages. There are other aspects of the voyage that involve the entire community, such as preparing special food and conditioning the body to withstand limited caloric intake during the voyage. Practitioners describe voyagers looking to travel between islands in the Pacific pre-transitioning to the diet they would expect out at sea several weeks in advance of the voyage.

Other activities require months or years of preparation, such as the building of the canoes and training of apprentice navigators and sailors. Still other forms of preparation require generational time, such as the growing and maintenance of certain trees to carve into the hulls of sailing canoes.

Most computational models of seafaring try to take into account data on canoe performance on other parameters, but in our metaphorical “preparing for the journey,” as we anticipate an exciting new wave of research, we place tremendous value on understanding the lived experiences of navigation practitioners from their perspective and in their own voices with the aim of developing strategies to incorporate some aspects of that cognitive, embodied and socially constructed knowledge into the computational models.

### Going to Sea

Once the choice to make a journey is set, and the voyage prepared, all that remains is to undertake the endeavor. Even with all the preparations made to maximise chances of success, there is no way to ensure that a voyage will go smoothly. After launch, success is heavily influenced by the navigator’s skills and the crew’s capabilities. Navigational practices, as shown above, vary by region and intention of the crew, knowledge and skill. These elements are strong inputs for model viability, as they are connected to the environmental factors they are based on and the start or end points of a journey mapped by algorithm. This is where seafaring theory connects not only to practice but more directly to computational modeling. However, it is reductive to say that these are the only elements of human choice or social influence that would have impacted voyaging. For example, if we consider routing, navigational practices can extend beyond choices made by a single seafarer to make the most of a current or passing wind. Factors such as the relationship between coastal, island and sea environments may have marked certain ports of call or areas of resource procurement or social or cosmological significance.

From a theoretical standpoint, as many of the methods for computational seafaring modeling are still evolving, how to incorporate these fundamental considerations into the process is a developing practice. The most evident example may be in how we explore the concepts of distance and time within models and in dissecting the viability of their outputs.

Whilst distance travelled is reliant on routing, technological capacity (for example ability to sail upwind) and environmental factors (weather, tides, currents), consideration of time allows for the inclusion of agency and experience. Common practice for seafaring modelers is to rely on time as the core cost expenditure for measuring the length of successful voyages. But comparing a straight value of hours may be undeserving of our complex understanding of how the concept of time functions within the seafaring process. Not only do we have to consider the time at sea, but the time to prepare for a voyage as well. It is clear that our consideration of the temporality of seafaring practice must shift to include these practices.

In addition, there is also the consideration of how perception may shift on what time means in the context of land versus marine environments (Farr 2010b; Leidwanger 2013). Whilst models may address these issues in bands of time marked by environmental pressures, the voyager may perceive the passage of time by the movement of celestial bodies or the cresting patterns of waves. When we consider the impacts of cognitive perceptions of time on human decision making, there may be more we need to evaluate concerning model outputs by measuring them not only against the feasibility of voyages that last a certain length of time but also how we might break down that time of the voyage into human-measured rather than algorithmic-measured intervals and thresholds. Looking for answers to this paradigm may lead us to ethnographic research, oral histories and current practitioner experience.

Beyond the passage of time at sea, it is important to consider the active art of navigation and its impact on both the length and location of voyages. Particularly in relation to navigation practices, and the variance in techniques used in models as well as real-world ‘practical’ scenarios, it is important to consider multiple frames of reference for seafaring. Oral histories or examples of direct, and indirect, communication of directions and experimental archaeology that reimagine these routes may help to build a foundation of understanding navigational practice in the deeper past. Reviewing what we can learn both from traditional and experimental practice is required for enhancing our ability to build effective models, as they may in turn impact the design of the algorithm that dictates movement across varied surfaces. It may be that talking directly with practitioners (such as those included in the CAST community) is a way to address this issue head on, and a way to help to validate end results for accuracy and realism. However, we must cautiously consider applying the practice of one region to another, one toolkit to another, or one mode of process to another. By comparing the expert account of practitioners across the globe, we may be able to begin to assess what is tied to environment or toolkit, which may help to define a better basis for models that can be applied globally versus those that are necessarily tied to specific uses (such as coastal or ocean-going parameters).

Experimental voyaging projects can aid us in understanding life at sea and are useful for testing physical (and emotional) stressors for travel (Hovarth and Finney 1969; Slayton 2018; see also Montenegro et al. 2023), currently an underexplored element of the field. However, we should note that practice between crews of the past and modern seafarers may not be directly comparable. Modern-day sailors using different techniques and skills based on learnt experience from within different (modern) vessels may make different choices, as would novice sailors who are interested in studying the past but come from non-maritime contexts. There is still work that needs to be done to evaluate comparisons between practitioner voyages, experimental voyages using reconstructed seafaring toolkits, and model outputs to determine where the boundaries between these types may lie and what impact knowledge of one may have on testing the viability of another.

Anthropology and cognitive science may also provide a background for us to discuss the effective practices of decision making, by helping computational modelers to better understand the influence of mental maps on seafaring practice. A distinction between communal and individual mental maps may also be useful for understanding variance in outcomes between those trips programmed to be run by experienced versus unfamiliar navigators. How much prior knowledge groups may have had of a route, or of the destination, impacted practical navigation concerns such as rough headings towards known navigational markers or harvestable resources.

Finally, it is important for modelers to understand the function of seascapes within the broader lives of those who used them in the past. Just as landscapes are active sites of engagement, exchange and creation, so too are maritime spaces. Seascapes were not static environments acting as a barrier or highway, but lived spaces that allowed for all manner of interaction. Indeed, it is useful for us to consider vessels as mobile sites. Beyond most current model considerations of time travelled, simple bands of access, or route locations, it is important for modelers to address ephemeral social and material elements of seascapes that may have influenced human action (see Shaikh 2022). This includes considering locations of resource procurement (such as mangroves or known fishing waters), non-static navigation aids (such as color change in water as directional markers, or tidal impacts on portages), points of longstanding symbolic importance (which may otherwise not register as obvious markers) and areas of avoidance (due to social conflict or participation, placement of known archaeological sites).

### Recounting the Voyage: Telling the Story of the Journey to the Ones Who Stay on the Shore

When a ship comes ashore, and especially when it returns safely to its home port, the contents of its cargo, its passengers and its crew become the vectors of a double narrative for those who remained on dry land. This narrative is, on the one hand, the basis for the development of a geography of the maritime and coastal spaces directly visited or perceived during the voyage and, on the other hand, an "imaginary" and sometimes mythical geography, the fruit of tales and products from more distant spaces and obtained through intermediaries encountered during the voyage: for example, Marco Polo’s (c. 1410–1412) description of Sypangu, Hanno the Carthaginian’s observations on West Africa’s maritime façade, or the land of the "women-without-men’’ from which Amazonian Indigenous peoples obtain their green rock pendants (Boomert 1987). These mythical and real topographies cannot be disentangled but serve to animate these spaces as dynamic and mark them as new human experiences. Meanwhile, the information upon return inscribes the sea—and coastal landscapes newly in view—into a wide range of different visual, oral and written representations. Moreover, all the information contained in this specific double narrative must take a form that allows it to be integrated into the general narrative, the reference system that underpins the society’s perception of the world and its own maritimity.

It is this loop of interaction between those who sail and those who stay ashore that we now turn, not in the context of ancient seafaring, but in the development of our computational approaches. How can we, after producing new knowledge about ancient maritime activity through computational approaches, integrate it into a global narrative about the maritimity of the groups concerned, for the benefit of the scientific community? And furthermore, what (conceptual) tools can and should we mobilise to build a narrative that can be appropriated by a wider public, especially by communities of descendants?

Since the development of numerical approaches to ancient navigation that go beyond simple drift models, the question of how to integrate data related to the navigational capabilities of ancient boats and ships, as well as those related to the skills and choices of the agents, has become central. This question concerns both the information gathered during dedicated experimental maritime archaeology projects, and that contained in historical sources and traditional oral accounts. A first set of data is relatively "easy" to collect and model. These are data on the load capacity, the route taken, and the speed of the boat in relation to the environmental conditions. This is true even if their "translation" from textual or oral sources requires prior dedicated historical or anthropological research (e.g. Casson 1951). The real difficulty arises when it comes to accounting for some of the biases that can strongly influence these data. The first of these is particularly relevant to information collected as part of experimental maritime archaeology programs (Bennett 2009) and to a lesser extent to that collected from contemporary maritime communities. This concerns the difference in skills that may exist between contemporary navigators and their ancient counterparts. The second concerns the consideration of crew capabilities during voyages due to the impact of various factors, in particular fatigue, hunger, emotional response or human relationships. Although numerical models can be developed for some of these factors (Montenegro et al. 2023), their translation into physical performance and decision making remains complex. Another distortion concerns the factors that can influence the decision-making process of crews, other than those related to technical and environmental elements. In fact, each society projects onto the physical realities of the sea a certain set of perceptions (i.e. risk/safety), values (positive or negative), rules and constraints, all of which form the basis of a real seascape, or even a real maritory. For those who traverse it, the sea is far from being a neutral space; it is full of places.

Transcribing the full complexity of the experience of seafarers into the development of numerical models poses a number of difficulties, with the return journey, from the computer to the sea deserving discussion.

In a manner of knowledge co-production, researchers can share and discuss results of simulations with traditional practitioners and experimental maritime archaeological projects to assess model success and accuracy. To achieve this, practitioners need to understand the general principles underlying the creation and operation of these digital models. For their authors, this means opening the black box by developing a specific narrative and, even more, by creating a veritable metalanguage around their practices. In particular, it is a matter of comparing the choices made by the machine with those made, or those that would have been made, by the practitioners in comparable navigations in the real world. The integration of this point of view, based on the agents’ perspective, allows us both to detect possible biases in the design of the digital model and to assess the distance that exists between the rational choices of the program and those of the actors, influenced by the perceptions, values, rules and constraints mentioned above. In addition to a better integration of anthropogenic factors, the aim is to use this exchange to motivate the joint design of new real-world experiments capable of producing the data needed to improve numerical simulations. The ultimate goal, then, is to create an interactive loop, a permanent navigation between ocean waves and electron flows.

## Conclusion

This paper provides a fresh perspective on seafaring theories that engage with practitioner knowledge to help the development and application of computational analysis discussed within this volume. The authors have focused on analysing the necessary interactions between the areas of anthropological theory, experimental or experiential approaches to seafaring research, ethnographic research and traditional knowledge. Written by specialists in different maritime regions, the authors provide unique but relevant examples of seafaring in the context of human agency, decision making, and social relations. These narratives are not intended to provide specifics for all maritime contexts globally, but rather to underline the value of understanding practice for the development of theoretical frameworks that can guide our evaluation of past seafaring.

Seafaring activities have played important roles in many traditional archaeological narratives. When archaeological research has focused predominantly on the preserved material culture of the past, seafaring activity is evident but intangible. This challenges us to find new ways to explore and understand this activity in terms of process, skill, knowledge and planning. Computational modeling is a useful tool to explore questions of seafaring in the past, but it is just that—a tool. We are not able to recount or recreate the past in an exact representation using these methods. Instead, the outputs from computational models are set to help us ask more questions and to guide our understanding about past voyages and seafaring traditions. As part of the iterative process of modeling as described by George Box in 1976, a feedback loop forms in which a combination of approaches link traditional archaeological and historical research with oceanographic and environmental data and experimental practice, whilst community engagement can guide how we construct our models, choose inputs and use the results. Our model outputs can then be evaluated through the lens of practice, run and re-run, to highlight multiple forms of engagement.

Through collaboration with traditional seafaring practitioners, modelers and archaeologists can better understand the range of maritime ontologies, the scope and context of maritimity and seafaring practices. Similarly, as seafaring is a social activity (see Helms 1988; Farr 2006) understanding the practices employed during voyages can teach us not just about boat technology, routes and distances traveled, but about how people experience and understand the world. Informed by new understanding of ways in which maritime space is encountered and created through collective action, the socially embodied processes of seafaring can be re-created through the digital medium of modeling.

When working within communities and with local practitioners to help shape our research questions and methods, it is also essential that we ask *why* and *how* this work can be useful concretely within these communities today. This falls within a growing movement to support community-led research, targeting research agendas and questions that are relevant and useful. Contemporary issues surrounding seafaring merge with debates around maritime migration and climate change that transcend the data from deep-time to that of present-day news. Environmental pressures, rising sea-levels, climate change, pollutants and changing hydrology result in shifting maritime practice and migration. Seafaring is a complex social process, but one that is deeply bound to the natural world. The boat itself functions as an interface, within and upon the ocean, touching sea and sky, whilst the seafarers and navigators possess profound knowledge and understanding of these environments. In many regions, this knowledge and skill has deep roots in the past. As such, understanding seafaring practice is also tied to understanding and documenting maritime heritage, central to many island and maritime communities around the world facing the loss of these traditions. With environmental change and sea-level rise, coastal development and modern maritime technologies—from GPS navigation systems to plastic roto-molded boats replacing traditional maritime technology and skills—the threat to both tangible and intangible heritage of seafaring is palpable. And as cultural heritage both tangible and intangible is linked to wellbeing and identity, these issues are timely and need to be addressed. It is hoped that through model co-creation and partnership with maritime communities, traditional seafaring practices can be documented and maritime heritages at threat can be shared, and in some way, preserved and promoted.

Computational modeling of voyages within changing marine environments are therefore *live* and iterative processes, part of the tradition of storytelling about the journey. They can be a useful tool to generate discussion, to highlight what can be mapped and modeled, and when used within a range of analytical tools, research practices and discussions, can help contextualise, preserve and extend our understanding of this aspect of maritime heritage.

## Data Availability

No datasets were generated or analysed during the current study.

## References

[CR1] Adams J (2013) A maritime archaeology of ships: innovation and social change in medieval and early modern Europe. Oxbow Books, Oxford

[CR2] Ammarell G (1999) Bugis navigation. Yale University Southeast Asia Studies, New Haven

[CR3] Arnold B (2018) Typologie et influence des bases monoxyles dans la construction navale traditionnelle, à l’image des esquifs réalisés par encorbellement inverse. Archaeonautica 20:165–182

[CR4] Barrena J, Harambour A, Lamers M, Bush S (2022) Contested mobilities in the maritory: implications of boundary formation in a nomadic space. Environ Plann c Polit Space 40:221–240

[CR5] Bennett J (2009) Sailing into the past: learning from replica ships. Naval Institute Press, Annapolis

[CR6] Bérard B, Biar A (2021) Indigenous navigation in the Caribbean Basin: a historical, ethnoarchaeological and experimental approach to the Caribbean-Guyanese kanawa. Archaeonautica L’archéologie Maritime Et Navale De La Préhistoire à L’époque Contemporaine 21:239–244

[CR7] Bérard B, Billard J, Letang T et al (2016a) Technologie du fait maritime chez les Kalinago des Petites Antilles au XVIe et XVIIe siècles. J De La Société des Américanistes 102–1:129–160

[CR8] Bérard B, Billard JY, Letang T et al (2016b) b. Approche expérimentale de la navigation précolombienne dans les Antilles. J De La Société des Américanistes 102:171–204

[CR9] Bérard B, Billard JY, Ramstein B (2011) Ioumoúlicou "*Koumoúlicou nhányem amonchéentium oúbao*" (Les Caraïbes qui viennent des autres îles sont gens de notre nation). In: Rebrovich S (ed) The Proceedings of the XXIII Congress of the International Association for Caribbean Archaeology, Antigua, 29 june-3 July 2009. Dockyard Museum, English Harbour, Antigua, pp 577–589

[CR10] Billard JY, Bérard B (2009) Apport de l’hydrostatique à l’archéologie expérimentale: Etude d’une pirogue de haute mer (Kanawa). 19th Congrès Français de Mécanique. Marseille

[CR11] Bird MI, Beaman RJ, Condie SA et al (2018) Palaeogeography and voyage modeling indicates early human colonization of Australia was likely from Timor-Roti. Quat Sci Rev 191:431–439. 10.1016/j.quascirev.2018.04.027

[CR12] Blankshein SL (2022) (Sea)ways of perception: an integrated maritime-terrestrial approach to modeling prehistoric seafaring. J Archaeol Method Theory 29:723–761. 10.1007/s10816-021-09536-4

[CR13] Blue L, Hocker F, Englert A (2003) Connected by the Sea: Proceedings of the 10th International Symposium on Boat and Ship Archaeology, Roskilde. Oxbow Books, Oxford

[CR14] Boomert A (1987) Gifts of the amazons: “green stone” pendants and beads as items of ceremonial exchange in Amazonia and the Caribbean. Anthropol 67:33–54

[CR15] Borreggine M, Powell E, Pico T et al (2022) Not a bathtub: a consideration of sea-level physics for archaeological models of human migration. J Archaeol Sci 137:105507. 10.1016/j.jas.2021.105507

[CR16] Box GE (1976) Science and statistics. J Am Stat Assoc 71:791–799

[CR17] Broodbank C (2013) The making of the Middle Sea: a history of the Mediterranean from the beginning to the emergence of the Classical World. Thames & Hudson, London

[CR18] Callaghan RT (2001) Ceramic age seafaring and interaction potential in the Antilles: a computer simulation. Curr Anthropol 42:308–313

[CR19] Casson L (1951) Speed under sail of ancient ships. Trans Am Philol Assoc 82:136–148

[CR20] Chadwick J (1990) The pech maho lead. Z Papyrol Epigr 82:161–166

[CR21] Cherry JF, Leppard TP (2015) Experimental archaeology and the earliest seagoing: the limitations of inference. World Archaeol 47:740–755

[CR22] D’Arcy P (2006) The people of the sea: environment, identity, and history in Oceania. University of Hawai’i Press, Honolulu

[CR23] Davenport W (1962) Red-Feather Money. Sci Am 206(3):94–105

[CR24] Davenport W (1964) Notes on Santa Cruz voyaging. J Polynesian Soc 73(2):134–142

[CR25] Dickson T, Farr H, Sear D, Blake J (2019) Uncertainty in marine weather routing. Appl Ocean Res 88:138–146. 10.1016/j.apor.2019.04.008

[CR26] Ditchfield K, Ulm S, Manne T et al (2022) Framing Australian Pleistocene coastal occupation and archaeology. Quat Sci Rev 293:107706. 10.1016/j.quascirev.2022.107706

[CR27] Farr H (2006) Seafaring as social action. J Marit Archaeol 1:85–99

[CR28] Farr H (2010b) b. Measurement in navigation: conceiving distance and time in the Neolithic. In: Morley I, Renfrew C (eds) The Archaeology of Measurement. Earth and Time in Ancient Societies, Cambridge University Press, Cambridge, Comprehending Heaven, pp 19–26

[CR29] Farr H (2010) a. Island colonization and trade in the Mediterranean. In: Anderson A, Barrett JH, Boyle KV (eds) The Global Origins and Development of Seafaring. McDonald Institute for Archaeological Research, Cambridge, UK, pp 179–189

[CR30] Feinberg R (1988) Possible prehistoric contacts between Tonga and Anuta. J Polynesian Soc 98:303–317

[CR31] Feinberg R (1991) A long-distance voyage in contemporary Polynesia. J Polynesian Soc 100(1):25–44

[CR32] Feinberg R, Genz J (2012) Limits of language for conveying navigational knowledge: way-finding in the Southeastern Solomon Islands. Am Anthropol 114(2):336–350

[CR33] Finney B (1985) Anomalous westerlies, El Niño, and the colonization of Polynesia. Am Anthropol 87:7–26

[CR34] Finney B (1986) Tracking Polynesian seafarers. Science 317:1873–1874 10.1126/science.1149035 17901319

[CR35] Finney B (1998) Nautical cartography and traditional navigation in Oceania. History Cartogr 2:443–494

[CR36] Fitzpatrick SM (2013) Seafaring capabilities in the pre-Columbian Caribbean. J Marit Archaeol 8:101–138

[CR37] Flatman J (2011) Places of special meaning: Westerdahl’s comet, agency, and the concept of the maritime cultural landscape. The Archaeology of Maritime Landscapes. Springer, New York, pp 311–329

[CR38] Fleury C (2013) The island/sea/territory. Towards a broader and three-dimensional view of the Aquapelagic Assemblage. Shima Int J Res Island Cult 7:1–13

[CR39] Ford B (2011) Coastal Archaeology. The Oxford handbook of maritime archaeology. Oxford University Press, Oxford, pp 763–785

[CR40] Genz J (2014) Complementarity of cognitive and experiential ways of knowing the Ocean in Marshallese navigation. Ethos 42:332–351

[CR41] Genz J (2016) Resolving ambivalence in marshallese navigation: relearning, reinterpreting, and reviving the “stick chart” wave models. Struct Dyn E J Anthropol Relat Sci 9:8–40

[CR42] Genz J (2018) Breaking the shell: voyaging from nuclear refugees to people of the sea in the Marshall Islands. University of Hawaiʻi Press, Honolulu

[CR43] Genz JH, Bardwell Jones C, Coffman M et al (2023) Voyaging in the pacific. Teach Ocean Seri Voyag Pacific 8:19–47

[CR44] George M (2012) Polynesian navigation and te lapa ‘the flashing.’ Time Mind J Arch Consciousn Cult 5(2):135–174

[CR45] Gladwin T (1970) East is a Big Bird. Harvard University Press, Cambridge, MA

[CR46] Gojda M (2001) Archaeology and landscape studies in Europe: approaches and concepts. In: One Land, Many Landscapes. British Archaeological Reports International Series 987, Archaeopress, Oxford, pp 9–18

[CR47] Hauʻofa E (1994) Our sea of islands. Contemp Pac 6(1):148–161

[CR48] Hauʻofa E (1998) The ocean in us. Contemp Pac 10:392–410

[CR49] Heidegger M (1993) Building, dwelling, thinking. In: Heidegger M (ed) Basic writings: martin heidegger. Routledge, London, pp 66–76

[CR50] Helms M (1988) Ulysses’ Sail: an ethnographic odyssey of power, knowledge and geographical distance. Princeton University Press, Princeton

[CR51] Hofman C, Borck L, Slayton E, Hoogland M (2019) Archaic age voyaging, networks, and resource mobility around the Caribbean Sea. Early settlers of the insular caribbean. Dearchaizing the Archaic. Sidestone Press, Leiden, pp 245–261

[CR52] Hofman C, Borck L, Lafoon J et al (2021) Island networks: transformations of inter-community social relationships in the Lesser Antilles at the advent of European colonialism. J Island Coast Archaeol 16:290–316

[CR53] Horvath SM, Finney BR (1969) Paddling experiments and the question of Polynesian voyaging. Am Anthropol 71:271–276

[CR54] Howe KR (ed) (2007) Vaka moana: voyages of the ancestors: the discovery and settlement of the pacific. University of Hawaiʻi Press, Honolulu

[CR55] Ingold T (1993) The temporality of the landscape. World Archaeol 25:152–174

[CR56] Ingold T (2021) The perception of the environment: Essays on livelihood, dwelling and skill. Routledge, London

[CR57] Irwin G (1992) The prehistoric exploration and colonization of the Pacific. Cambridge University Press, Cambridge

[CR58] Irwin G (2007) Voyaging and Settlement. Vaka moana: voyages of the ancestors: the discovery and settlement of the pacific. University of Hawaiʻi Press, Honolulu, pp 55–91

[CR59] Irwin G, Bickler S, Quirke P (1990) Voyaging by canoe and computer: experiments in the settlement of the Pacific Ocean. Antiquity 64(242):34–50. 10.1017/S0003598X00077280

[CR60] Jarriel K (2018) Across the surface of the sea: maritime interaction in the Cycladic Early Bronze Age. J Mediterr Archaeol 31:52–76. 10.1558/jma.36810

[CR61] Kealy S, Louys J, O’Connor S (2017) Reconstructing palaeogeography and inter-island visibility in the Wallacean Archipelago during the likely period of Sahul colonization, 65–45 000 years ago. Archaeol Prospect 24:259–272

[CR62] Kirch PV (2017) On the road of the winds: an archaeological history of the Pacific Islands before European contact. Univ of California Press, Oakland

[CR63] Kuijjer E, Haigh I, Marsh R, Farr H (2022) Changing tidal dynamics and the role of the marine environment in the maritime migration to Sahul. PaleoAnthropology 2022:134–148

[CR64] Leidwanger J (2013) Modeling distance with time in ancient Mediterranean seafaring: a GIS application for the interpretation of maritime connectivity. J Archaeol Sci 40:3302–3308

[CR65] Lejeune M, Pouilloux J (1988) Une transaction commerciale ionienne au Ve s. à Pech-Maho. CRAI 3:526–536

[CR66] Lewis D (1994) We, the navigators: the ancient art of landfinding in the Pacific. University of Hawai’i Press, Honolulu

[CR67] McGrail S (2009) Experimental archaeology: replicas and reconstructions. In: Bennet J (ed) Sailing into the past. Seaforth Publishing, Barnsley, Learning from replica ships, pp 16–23

[CR68] Molle G, Hermann A (2018) Pitcairn before the mutineers: revisiting the isolation of a Polynesian Island. The Bounty from the beach: cross-cultural and cross-disciplinary essays. ANU Press, Canberra, pp 67–94

[CR69] Montenegro A, Niclou A, Anderson A et al (2023) Estimated energetic demands of thermoregulation during ancient canoe passages from Tahiti to Hawaii and New Zealand, a simulation analysis. PLoS ONE 18:e0287290. 10.1371/journal.pone.0287290 37437072 10.1371/journal.pone.0287290 PMC10337932

[CR70] Muckelroy K (1978) Maritime archaeology. Cambridge University Press, Cambridge

[CR71] Needham S, Clark P (2009) Encompassing the Sea: maritories and Bronze age maritime interactions. Bronze Age connections: cultural contact in prehistoric Europe. Oxbow Books, Oxford, pp 12–37

[CR72] Norman K, Inglis J, Clarkson C et al (2018) An early colonization pathway into northwest Australia 70–60,000 years ago. Quat Sci Rev 180:229–239. 10.1016/j.quascirev.2017.11.023

[CR73] Marco Polo (1410) Le Livre des Merveilles du Monde

[CR74] Pomey P (2013) Defining a ship: architecture, function, and human space. In: Ford B, Hamilton DL, Catsambis A (eds) The Oxford handbook of maritime archaeology. Oxford University Press, Oxford, pp 25–56

[CR75] Pyrek C, Feinberg R (2016) The vaeakau-taumako wind compass as part of a ”navigational toolkit”. Struct Dyn 9(1):41–69

[CR76] Ravn M, Bischoff V, Englert A, Nielsen S (2013) Recent advances in post-excavation documentation, reconstruction, and experimental maritime archaeology. In: Ford B, Hamilton DL, Catsambis A (eds) The Oxford handbook of maritime archaeology. Oxford University Press, Oxford, pp 232–249

[CR77] Schück A (1902) Die Stabkarten der Marshall-Insulaner. Kommissionsverlag von H.O. Persiehl, Hamburg

[CR78] Sear D, Allen M, Hassall J et al (2020) Human settlement of East Polynesia earlier, incremental, and coincident with prolonged South Pacific drought. Proc Natl Acad Sci U S A 117:8813–8819 32253300 10.1073/pnas.1920975117 PMC7183181

[CR79] Shaikh ZA (2022) Navigating the Red Sea: A spatiotemporal approach to investigating 18^th^-19^th^ century CE Indigenous seafaring. University of Southampton, doctoral thesis

[CR80] Slayton ER (2018) Seascape corridors: modeling routes to connect communities across the Caribbean Sea. Sidestone Press, Leiden

[CR81] Steinberg P, Peters K (2015) Wet ontologies, fluid spaces: giving depth to volume through oceanic thinking. Environ Plann D Soc Space 33:247–264

[CR82] Tamagyongfal S, Myazoe J, Genz J (2023) Post-settlement voyaging networks of Yap and the Marshall Islands: Examples of ancestral adaptive capacity in response to environmental changes and disasters. Mains’l Haul A J Pacif Marit History 58(1–4):48–56

[CR83] Teaiwa TK (2005) The classroom as a metaphorical canoe: cooperative learning in pacific studies. WINHEC Int J Indigen Edu Schol 1:38–48

[CR84] Thomas S (1987) The last navigator. Ragged Mountain Press, Camden, International Marine

[CR85] Tilley C (2004) The materiality of stone: explorations in landscape phenomenology. Berg, Oxford

